# Field-Portable Leukocyte Classification Device Based on Lens-Free Shadow Imaging Technique

**DOI:** 10.3390/bios12020047

**Published:** 2022-01-18

**Authors:** Dongmin Seo, Euijin Han, Samir Kumar, Eekhyoung Jeon, Myung-Hyun Nam, Hyun Sik Jun, Sungkyu Seo

**Affiliations:** 1 Department of Electronics and Information Engineering, Korea University, Sejong 30019, Korea; dseo@kriso.re.kr (D.S.); haan919@korea.ac.kr (E.H.); skumar@korea.ac.kr (S.K.); 2 Maritime Safety Research Division, Korea Research Institute of Ships & Ocean Engineering, Daejeon 34103, Korea; 3 Department of Biotechnology and Bioinformatics, Korea University, Sejong 30019, Korea; uuu8345@korea.ac.kr; 4 Department of Laboratory Medicine, College of Medicine, Korea University Anam Hospital, Seoul 02841, Korea; yuret@korea.ac.kr

**Keywords:** lens-free shadow imaging technique (LSIT), CMOS image sensor, leukocytes, diagnostics, blood cell classification

## Abstract

The complete blood count (CBC) is one of the most important clinical steps in clinical diagnosis. The instruments used for CBC are usually expensive and bulky and require well-trained operators. Therefore, it is difficult for medical institutions below the tertiary level to provide these instruments, especially in underprivileged countries. Several reported on-chip blood cell tests are still in their infancy and do not deviate from conventional microscopic or impedance measurement methods. In this study, we (i) combined magnetically activated cell sorting and the differential density method to develop a method to selectively isolate three types of leukocytes from blood and obtain samples with high purity and concentration for portable leukocyte classification using the lens-free shadow imaging technique (LSIT), and (ii) established several shadow parameters to identify the type of leukocytes in a complete leukocyte shadow image by shadow image analysis. The purity of the separated leukocytes was confirmed by flow cytometry. Several shadow parameters such as the “order ratio” and “minimum ratio” were developed to classify the three types of leukocytes. A shadow image library corresponding to each type of leukocyte was created from the tested samples. Compared with clinical reference data, a correlation index of 0.98 was obtained with an average error of 6% and a confidence level of 95%. This technique offers great potential for biological, pharmaceutical, environmental, and clinical applications, especially where point-of-care detection of rare cells is required.

## 1. Introduction

The complete blood count (CBC) is a routine laboratory test used in clinical medicine for the early detection of serious diseases [1,2]. A CBC measures the size, quantity, and maturity of the various blood cells in a blood sample. Erythrocytes, leukocytes, and platelets are the major components of human blood [3]. Erythrocytes, which make up most of the blood cells, transport oxygen and carbon dioxide to and from tissues. Leukocytes, responsible for the defense mechanisms of the body, are signs of infection or disease and provide superficial information on the health of the patient. Platelets are colorless cells that form blood clots and prevent bleeding. Leukocytes are divided into five subtypes, most of which are neutrophils, lymphocytes, and monocytes. Neutrophils, which account for more than 40–70% of leukocytes, are produced primarily during bacterial infections and diseases and respond rapidly to chemotaxis [4]. Lymphocytes, which comprise approximately 30% of leukocytes, are immunoreactive cells and are further subdivided into T cells, B cells, and natural killer cells (NK). B cells produce antibodies, and NK cells kill infected and cancer cells as part of the innate immune response [5]. The third type of leukocyte is the monocyte, which represents approximately 3–5% of all leukocytes and ingests foreign bodies or dead cells in the human body. Since leukocytes react differently depending on their type and action, leukocyte count is clinically crucial as the primary diagnosis.

There are numerous manual and automated methods for the examination of human blood cells. Traditional manual methods for blood cell classification such as morphological profiling of blood cells by manual smear and visual inspection are labor intensive and do not provide quality control. In addition, the manual technique has disadvantages such as observer bias, slide distribution problems, statistical sampling and recording errors, and the need for highly skilled technicians [6,7]. Automated approaches such as the Coulter method (based on electrical impedance) and fluorescence-activated cell sorting (FACS) have been developed to overcome these limitations [8,9,10,11]. Although these automated techniques allow for the rapid analysis of large numbers of samples, they have several limitations including the need for heavy, expensive, and powerful equipment and skilled personnel [12]. Therefore, these facilities are accessible only to high-level medical facilities. Recently, several point-of-care (POC) systems have been proposed to assess blood count with high precision to overcome these drawbacks. Microfluidics-based blood analysis systems that either perform fluorescence analysis or combine magnetic purification of cells with chemotaxis assay from small blood samples have been reported [13,14,15,16,17]. However, they all have accessibility limitations and require additional fluorescence analysis to identify the class and concentration of fluorescently stained cells or additional fluorescence staining to confirm the pathway of cell migration. Recently, much interest has been expressed in the development of systems to automatically classify digital images of peripheral blood smears with high sensitivity and specificity [18,19,20,21]. Many groups have reported leukocyte classification of microscopic blood images using an adaptive neuro-fuzzy inference system or leukocyte classification based on spatial and spectral features of microscopic hyperspectral images [22,23,24]. However, these technologies use high-quality optical microscopes or lenses, which limits their application to low-cost, portable platforms for on-site cell monitoring.

**Figure 1:**
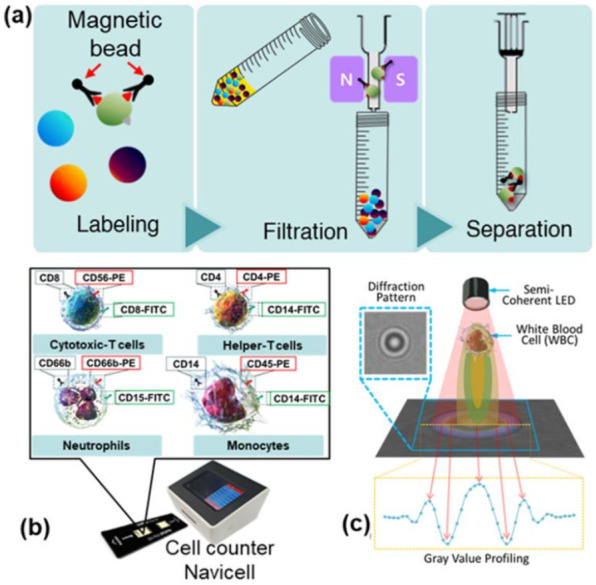
(**a**) Schematic representation of the magnetically activated cell sorting technique (MACS) for selective detection of specific cells in blood using a magnetic nanoparticle conjugated with antibodies. MACS is a three-step process that includes labeling, filtration, and separation. (**b**) An exterior view of the device along with the diagnostic chip. (**c**) Schematic of the lens-free shadow imaging system.

In contrast, the lens-free shadow imaging technique (LSIT) is emerging as a potential tool to replace expensive microscope-based cell analysis [25]. Instead of examining the original cell image, LSIT takes the diffraction pattern of a cell and examines its properties to determine the cell type. When light from a semi-coherent light-emitting diode (LED) is irradiated through a micro-pinhole, the light can be scattered in three ways: It can penetrate the cell, scatter from the cell surface, and cannot reach the cell (Figure 1) [26]. A diffraction pattern on a CMOS image sensor represents the interference of these three forms of light. LSIT consists of only two optoelectronic elements (LED and CMOS image sensors) and does not require any optical structures or lenses, eliminating the entire process of optical focusing. Therefore, it has the advantage of low manufacturing cost and compact design [27]. Moreover, it is a high-throughput technique with a wide field of view, which is approximately ten times greater than the measurement range of a hemocytometer under an optical microscope [28]. We have previously reported that heterogeneous solutions of erythrocytes, yeast cells, *E. coli*, and microparticles of different sizes can be characterized automatically without lenses or microscope objectives. LSIT can be used to determine hemoglobin concentration [29,30], analyze the growth of bacterial biofilms [31], and perform various cell counts [27,32]. LSIT has advantages such as compactness, low cost, and high throughput because it does not require structures for optical lenses and alignments [25]. We have also previously reported the development of a lens-free imaging platform for the stain-free measurement of cell viability and automated counting of blood cells [27,33]. In that study, a cell lysis protocol was used before leukocyte detection. Erythrocyte lysis is commonly performed as part of the processing of blood samples for immunophenotyping by flow cytometry. The reduction or removal of erythrocytes by lysing facilitates leukocyte testing. However, lysing erythrocytes have numerous implications for immunophenotyping including potential cell loss. Furthermore, this technique requires a homogeneous sample to create a shadow image library for the corresponding sample.

This study describes a technique for classifying three types of leukocytes using LSIT and creating a shadow image library for each cell type. By combining magnetically activated cell sorting (MACS) and the differential density method, we have developed, for the first time, a method to selectively isolate three types of leukocytes in blood and obtain samples with high purity and concentration for the classification of leukocytes with LSIT. We then established several shadow parameters to identify the type of leukocyte in a whole-shadow leukocyte image by shadow image analysis. Finally, we demonstrate the accuracy and performance of the proposed shadow image parameters. Our NaviCell device costs ~$500 in material base, which is far less than the expensive (>$200K) fully automated, bulky, and complicated system that requires well-trained operators and a lab. Furthermore, it costs almost eight times less (>$4000) than the commercial benchtop cell counters, which have the disadvantage of high error rate and low throughput [27]. Thus, this low-cost and small blood analysis platform can be used as a powerful point-of-care (POC) diagnostic tool in the field, especially in resource-limited environments.

## 2. Materials and Methods

### 2.1. Human Whole Blood Preparation

Whole blood samples were collected from 20 outpatients at Anam Hospital, Korea University, with approval from the Institutional Review Board (# 2021AN0040). Clinical examination of these samples showed that blood cells were within the normal range (neutrophils: 40–70%; lymphocytes: 15–40%; monocytes: 0–15%). Blood was collected in an anticoagulant tube and stored at 4 °C until further use. Cell separation buffer was prepared by adding 0.5% bovine serum albumin (BSA; Bovogen Biologicals, Keilor East, Australia) and 2 mM ethylenediaminetetraacetic acid (EDTA; LPS Solution, Daejon, Korea) to Dulbecco’s phosphate buffered saline (DPBS; Merck KGaA, Darmstadt, Germany). BSA serves as a buffer preservative and prevents nonspecific antibody binding in the sample [34].

#### 2.1.1. Magnetically Activated Cell Sorting of Human Whole Blood

MACS is a technique for the selective detection of specific cells in the blood using a magnetic nanoparticle conjugated with antibodies specific for the cell surface proteins of the corresponding leukocytes (Figure 1a) [35]. MACS was conducted via a three-step procedure including labeling, filtration, and separation, as described in the instruction manual (Miltenyi Biotec, Bergisch Gladbach, Germany). Single cells were prepared by passing them through a 20 µm cell filter (Fisher Scientific, Waltham, MA, USA) before MACS. Briefly, target cells of interest were labeled with a specific antibody conjugated magnetic nanoparticle against a cell surface marker (Figure 1a) at 4 °C for 20 min in the dark. After the reaction was completed, the labeled cells were separated from the unlabeled cells by passing the entire mixture through the magnetic field. Consequently, the labeled cells were attached to the filter system while the unlabeled cells were filtered (flow-through). After five consecutive washes with the separation buffer, the labeled target cells were eluted from the filter by removing the magnetic field and adding the separation buffer. Neutrophils were first separated from whole blood using MACS and flow through containing erythrocytes, peripheral blood mononuclear cells (PBMC), and granulocyte remnants was subjected to a further isolation process.

#### 2.1.2. Cell Separation Using a Density Gradient Medium (Ficoll Solution)

For further isolation, density gradient centrifugation with Ficoll solution (Ficoll-PaqueTM Plus; Merck KGaA, Darmstadt, Germany) was used to separate lymphocytes and monocytes. The Ficoll solution (a low-viscosity, high-density epichlorohydrin chemical) uses density differences to separate blood samples into plasma, peripheral blood mononuclear cells (PBMCs), granulocytes, and erythrocytes. First, 4 mL of blood sample (diluted 1:1 with DPBS) was slowly poured into 3 mL of Ficoll solution to form a layer. The mixture was separated into four layers by centrifugation at 400× *g* for 30 min. As a result, the densest erythrocytes and granulocytes were in the bottom layer, followed by the layer containing the Ficoll solution, then the thin and opaque stained PBMC layer, and finally the platelets and plasma in the top layer. The PBMC layer was separated and centrifuged at 100× *g* for 10 min. The supernatant was discarded and highly pure mononuclear cells were yielded.

Because the PBMC layer contains both lymphocytes and monocytes, the MACS approach described in Section 2.1.1 was used to separate these two cell types. The MACS approach can theoretically separate lymphocytes and monocytes from whole blood. However, because monocytes are present in low concentrations in blood, separating erythrocytes and neutrophils from the sample is complicated. Therefore, the combination of density gradient centrifugation and MACS provides a powerful tool for obtaining a high yield of homogeneous leukocytes. For a schematic representation of the MACS protocol for lymphocyte separation, see Figure S1.

### 2.2. Experimental Setup

#### 2.2.1. No-Stain and Automated, Versatile, Innovative Cell Analysis (NaviCell)

Shadow images of three types of leukocytes (neutrophils, lymphocytes, and monocytes) were obtained using NaviCell with the LSIT described in Section 1. Figure 1b,c shows the NaviCell cell counter used for this study and a schematic of the lens-free cell imaging system, respectively. More details on the NaviCell device can be found elsewhere [24]. The cell counter consisted of a blue LED (450–490 nm; Harvatek Co. Ltd., Hsinchu, Taiwan) to take advantage of the high-contrast diffraction patterns and a CMOS image sensor (MT9P031; Micron Technology, Manassas, VA, USA) with a sensing area of 24.396 mm^2^. A 300 ± 5 μm (K39-880; Edmund Optics, Barrington, NJ, USA) pinhole was used on top of the LED to achieve semi-coherent illumination. On the top of the cell counter was a 5-inch touch display for quick confirmation of the measured image. On the front of the cell counter is a connector for the cell chip, while the back contains a USB port and power switch. These components were fully packed into the NaviCell device, which measures 16.2 × 13.5 × 13.8 cm. A black disposable chip was used for the measurements to minimize interference from external light, and only the area where the sample was measured was transparent. This custom chip measures 7.5 × 2.2 cm, and the area with two detectors can hold up to 10 μL of sample. After adding the 10 μL sample to each of the two detection units, the chip was inserted into the cell chip port of the cell counter, and the measurements were collected and displayed on a 5-inch touch display via an Android-based user interface. The shadow image of the sample and the measurement result can be quickly and visually confirmed via the display. The measurement result, measurement time, and information about the measurement cell can be stored on the memory of the cell counter and backed up externally via the USB drive.

#### 2.2.2. Correlation, Linearity, and Agreement

Two reference systems were used to verify the techniques used in this study. First, a Coulter counter (DxH 800 Hematology Analyzer, Beckman Coulter, Indianapolis, IN, USA) was used to obtain the reference CBC data for the blood samples used in the analysis. Personal identity data such as names and addresses are protected by law and information regarding the individuals’ health and financial circumstances are protected. This CBC result was used as comparative data to verify the accuracy of the parameters developed for shadow image analysis. Flow cytometry (Guava easyCyte 8-10HT, Millipore, Burlington, MA, USA) was used to determine the purity of the isolated leukocyte samples. Since flow cytometry can distinguish types of blood cells using fluorescent antibodies, it is possible to confirm the purity of the separated leukocytes. The results of this analysis confirmed that high purity and high concentration leukocyte samples were obtained.

#### 2.2.3. Shadow Parameters

The NaviCell described in Section 2.2.1 was used to collect shadow images of the three types of leukocytes, and a library was created. To determine the characteristics of these shadow images of the three types of leukocytes, 100 shadow images of each type of cell were extracted and the intensity of the pixels was secured using the profile function of the ImageJ program (NIH). By comparing the obtained intensities, the basic parameters for the specific discrepancies were determined and integrated. With these basic parameters, the three types of leukocytes (i.e., neutrophils, lymphocytes, and monocytes) could be distinguished in the shadow image. For cell classification, the central maximum value (CMV), the first-order minimum value (MIN), and the first-order maximum value (MAX) were extracted. The difference between the CMV and MIN values was defined as the peak-to-peak distance (PPD) and the difference between the CMV and MAX values was defined as the maximum-to-minimum distance (MMD). In this study, it was difficult to distinguish helper T cells from cytotoxic T cells because all basic parameter values except CMV were similar. However, for lymphocyte type, they were different from the neutrophils and monocytes. By combining the values of the above basic parameters, the shadow image parameters were developed to distinguish the three types of leukocytes. The order ratio (*OR*), defined as the ratio of *PPD* to *MMD*, was introduced as a metric to distinguish neutrophils from leukocytes, given by

(1) $$OR=\frac{PPD}{MMD}$$

The minimum ratio (*MR*), defined as

(2) $$MR=\frac{MIN}{MMD}$$

was proposed as a metric to distinguish lymphocytes from the remaining mononuclear cells after neutrophils were preferentially filtered on the leukocyte shadow image using the *OR* parameter.

### 2.3. Detection Algorithm

The leukocyte shadow images acquired with NaviCell were processed with a program specifically designed for blood cell analysis. The original image to be analyzed was duplicated to produce two identical images. After the background signal was extracted from the first image, binarization was performed. The feature points of the binarized cells were then grouped using clustering to create an image without a background. The grouped pixels were recognized as one cell, and the median of the group was stored as the median of the cell. The cells were then recognized by merging them with the images from the second copy. Finally, the results of the blood analysis were determined using the values of the essential parameters. The software was able to examine hundreds of cells in one image within a minute [23]. To measure the performance of the developed *OR* and *MR* parameters for leukocyte identification, the *F*1 score was calculated. The *F*1 score is the harmonized mean of *Precision* and *Recall*, where

(3) $$Precision=\frac{TP}{TP+FP}\;$$

(4) $$Recall=\frac{TP}{TP+FN}$$

(5) $$F1=2\times \frac{Precision\times Recall\;}{Precision+Recall}$$

*TP*, *FP*, and *FN* are the true positives (sensitivity), false positives (specificity), and false negatives, respectively. *Precision* is the number of data that the model identifies as ‘true’ and *Recall* is the number of data that the model perceives as ‘true’. The *F*1 score is an indicator of the effectiveness of the model, with an *F*1 score reaching its best value at 1 and its worst value at 0.

## 3. Results and Discussion

**Figure 2:**
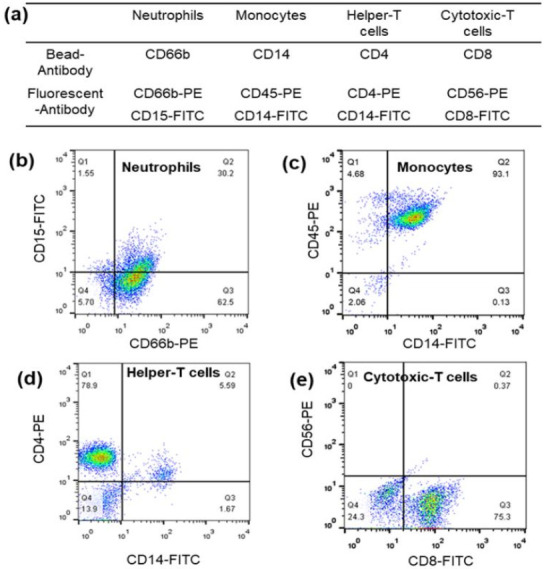
(**a**) Various antibodies conjugated to magnetic beads for whole blood leukocyte isolation and fluorescent antibodies. Flow cytometric analysis of the purity of (**b**) neutrophils, (**c**) monocytes, (**d**) helper T cells, and (**e**) cytotoxic T cell leukocyte samples obtained by a combination of magnetically activated cell sorting (MACS) and the differential density method.

Neutrophils, lymphocytes, and monocytes were selectively isolated with high purity using a combination of magnetic bead separation and density gradient centrifugation. The shadow image confirmed the homogeneous cells, and the magnified shadow images showed a uniform overall appearance for all three cells, as shown in Figure S2. The exact purity of the samples was validated using fluorescent antibodies attached to the cells and analyzed by flow cytometry. Various antibodies conjugated to magnetic beads for the isolation of whole blood leukocytes and fluorescent antibodies are shown in Figure 2a. The purity of neutrophils, monocytes, helper T cells, and cytotoxic T cells was determined to be 93%, 93%, 79%, and 75%, respectively (Figure 2b–e).

Various antibodies and magnetic beads can affect leukocyte shadow images. The application of the developed parameters that depend on the shadow images would be problematic if the magnetic beads altered the shadow images. Therefore, the effect of antibodies or magnetic beads on the shadow images of leukocytes was also investigated by comparing the shadow images of leukocytes separated with magnetic beads with those of the control samples. To confirm the effect of different antibodies on cells and their shadow images, mononuclear cell samples mixed with lymphocytes and monocytes (obtained with Ficoll solution) were used as a control group and the shadow images of the lymphocytes and monocytes separated with magnetic beads were compared for each cell type. We have already noted that the high erythrocyte concentration in human blood (~4 × 10^6^ cells/μL) is quite a high concentration for the FOV of 24.396 mm^2^ in a shadow imaging system [27]. However, the probability of cell overlap is relatively low due to the uneven distribution of cells, which limits the accuracy of cell counting using the shadow image technique (about 6% for 400 cells in the FOV of 24.396 mm^2^ at a dilution factor of 16,000 for erythrocytes) [27]. Due to the 1000-fold lower concentration of leukocytes compared to erythrocytes, the possibility of cell overlap is extremely low, even with a slightly higher dilution factor.

**Figure 3:**
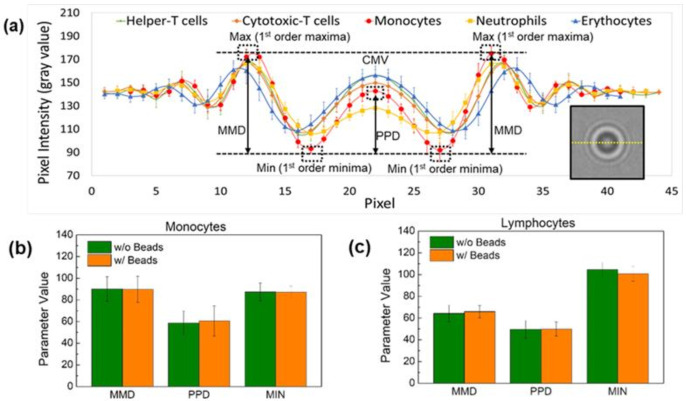
(**a**) Schematic representation of the parameters extracted to distinguish the three types of leukocytes—neutrophils, lymphocytes, and monocytes—on the shadow image. Comparison of the effect of magnetic beads on the parameters MMD, PPD, and MIN of (**b**) monocytes and (**c**) lymphocytes. The difference between the central maxima value (CMV) and the first-order minima value (MIN) is defined as the peak-to-peak distance (PPD) and the difference between the CMV and the first-order value (MAX) is defined as the maxima-to-minima distance (MMD). The presence or absence of magnetic beads has no apparent effect on the shadowing of the cells.

The baseline parameters MMD, PPD, and MIN were used to compare cells in the control and comparison groups (Figure 3a). The percentage errors of the MMD, PPD, and MIN values for lymphocytes with and without magnetic beads were 2.5%, 0.58%, and 3.75%, respectively (Figure 3b,c). Additionally, the percentage errors for each parameter were 0.41%, 3.39%, and 0.16% for monocytes with and without magnetic beads, respectively. Thus, it can be concluded that the presence or absence of magnetic beads has no apparent effect on the shadow image of the cells.

**Figure 4:**
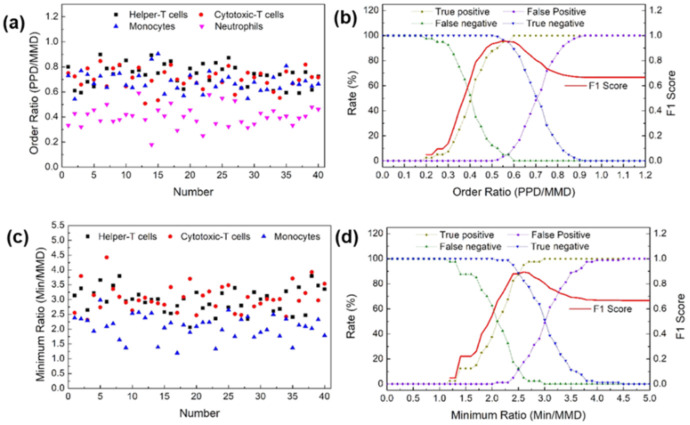
(**a**) Order ratios (ORs) for major leukocyte subpopulations. (**b**) F1 score to distinguish between neutrophils and leukocytes using the OR. (**c**) Minimum ratios (MRs) for major leukocyte subpopulations. (**d**) F1 score to distinguish between lymphocytes and the rest of the mononuclear cells using the MR.

Figure 4a,c shows the OR and MR distribution of the leukocyte subpopulations based on shadowing characteristics. Neutrophils had an OR distribution of 0.6 or less. This value is different from the distribution of the other cells. The distribution of MR by lymphocyte and monocyte types in mononuclear cells was confirmed. In lymphocytes, the distribution ranged from 2.5 ≤ MR < 4.5, and in monocytes, the distribution ranged from 1 ≤ MR < 2.5. Therefore, lymphocytes can be distinguished from monocytes with a MR distribution of less than 2.5. However, similar to the profile profiling results, helper T cells and cytotoxic T cells with the same MR range were set as one lymphocyte type. The F1 score was calculated based on the parameters OR and MR. Figure 4b,d shows the F1 score for identifying neutrophils and distinguishing lymphocytes from monocytes, respectively. The highest F1 score of 0.96 for neutrophil identification was obtained at an OR value of 0.55. The best F1 score for lymphocytes was 0.89, with an MR value of 2.6. Using this method, we can quickly determine the best OR MR value to classify leukocytes.

Blind tests were performed on blood samples from 20 randomly selected patients to evaluate the accuracy and reliability of the proposed OR and MR parameters for the classification of the three leukocyte types. Leukocyte samples were prepared by selectively labeling magnetic beads with CD45 antibodies. Then, shadow images of the leukocytes were made using the NaviCell counter, and the parameters OR and MR were applied to the data. The ratios of the three primary leukocyte subtypes were classified according to each shadow parameter and compared with the 20 CBC data from Anam Hospital, Korea University. The difference measurements for the 20 samples measured with CMOS and the reference system are summarized in Table 1.

The average error rates for neutrophils and lymphocytes were approximately 2% and 3%, respectively, while the error rate for monocytes was approximately 14%; however, with the exception of two monocyte samples that had error rates greater than 100%, the average error rate for monocytes was approximately 3.9%. Blood samples from patients who requested a blood test were negative for disease or infection. Thus, if the shadow parameter classifies the diffraction pattern on the cell surface, the change in monocyte response to disease is not obvious and the inaccuracy could be due to multiple variables. Furthermore, because the leukocyte ratio is small, a deviation of one minute would significantly affect the ratio. The average neutrophil, lymphocyte, and monocyte populations calculated with the lens CMOS system for the 20 samples were 59.20%, 27.60%, and 10.39%, respectively. This differential result agrees well with the measurement of the reference system (58.50%, 28.57%, and 9.14%, respectively), with a correlation index of 0.999 between the two modalities.

**Figure 5:**
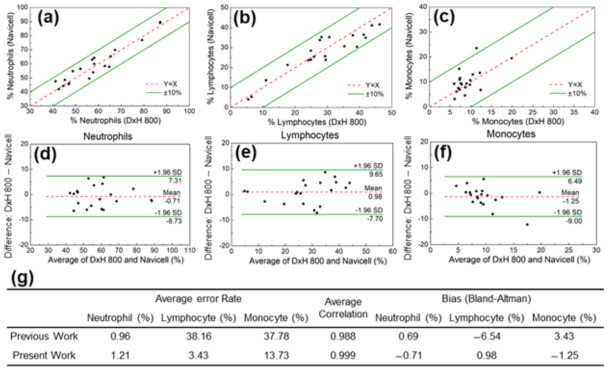
Comparison of (**a**) neutrophil, (**b**) lymphocyte, and (**c**) monocyte populations measured with the CMOS system and the reference system (DxH 800 hematology analyzer, Beckman Coulter). Bland–Altman analysis for (**d**) neutrophil, (**e**) lymphocyte, and (**f**) monocyte populations. (**g**) The average error rate for the classification of neutrophils, lymphocytes, and monocytes in the present and our previously reported method.

The effectiveness of the proposed method was evaluated by examining the detection accuracy of each leukocyte subtype. The neutrophil ratio obtained by applying the OR parameter to the shadow image was compared with the reference CBC data from the Korea University Anam Hospital. Direct comparison of the two approaches for neutrophils yielded a mean error of 1.21%, with all 20 data points within the 10% error limit (Figure 5a). Similarly, a mean error of 3.4% and 13.7% was obtained for lymphocytes and monocytes, respectively (Figure 5b,c). Using the shadow parameters developed in this study, the average error rate for the classification of neutrophils, lymphocytes, and monocytes was better than that of our previously reported method (Figure 5g) [32]. Furthermore, the average correlation was 0.999, which was also much better than in the previous study.

The Bland–Altman method was used to demonstrate the reliability of the OR image parameter in identifying neutrophils in the leukocyte shadow image and to evaluate the agreement between the two systems. As shown in Figure 5d, the analysis revealed a bias of −0.71%, with 95% limits of agreement ranging from 7.31% to −8.73%. The same analysis was performed for the other leukocyte subgroups. The results for lymphocytes (bias 0.98%, with 95% limits of agreement between 9.65% and −7.70%) and monocytes (bias −1.25%, with 95% limits of agreement between 6.49% and −9.00%) are shown in Figure 5e,f.

## 4. Conclusions

In summary, by combining the MACS and density gradient centrifugation methods, a method for purification of neutrophils, lymphocytes, and monocytes from human whole blood was developed to identify and classify shadowgraphs of three types of leukocytes in whole blood using LSIT. The LSIT-based NaviCell counter can collect information on individual cells from hundreds of cells in a minute. The presence or absence of magnetic beads did not affect the diffraction pattern. From the samples obtained by these methods, we created a shadow image library of the three leukocyte subtypes. The shadow parameters, OR and MR, were developed by combining different features classified according to the type of cells in the shadow images of the library. The OR can separate neutrophils from leukocyte shadow images, while MR can distinguish lymphocytes from monocytes. The ratios of the three types of leukocytes obtained with the defined parameters were compared with the reference CBC data from 20 patients. The error rates for neutrophil count, lymphocyte count, and monocyte count were 2.5%, 3.8%, and 14.2%, respectively, with an overall reliability of 95%. Subsequently, a high correlation coefficient of 0.98 was confirmed with the reference data. Advances in computer power and algorithm development have changed the paradigm of how machines learn from data. Deep learning is a paradigm that can describe data and learn independently at different levels of abstraction using a model composed of numerous levels of processing, rather than human-defined rules. In conjunction with LSIT, this technology enables cell separation and analysis at the whole blood level without antibodies. In addition, the method is expected to be able to evaluate previously indistinguishable images such as those of helper and cytotoxic T cells. Finally, we can envision applications in various areas of cell analysis including smart cell detection and malaria detection. This finding has immense potential for the development of point-of-care and wireless health applications, especially in resource-constrained environments.

## Figures and Tables

**Table 1:** Three-part differential WBC counts for 20 outpatient blood samples measured by the NaviCell counter and the reference system (DxH 800 Hematology Analyzer, Beckman Coulter).

Sample	Method	Neutrophil (%)	Lymphocyte (%)	Monocyte (%)	Correl.	Sample	Method	Neutrophil (%)	Lymphocyte (%)	Monocyte (%)	Correl.
1	DxH 800	57.70	28.20	9.80	0.973	11	DxH 800	47.10	37.90	12.20	0.999
	NaviCell	54.01	35.31	8.9			NaviCell	46.21	36.22	13.70	
2	DxH 800	64.80	24.90	7.30	0.991	12	DxH 800	52.10	24.90	19.90	0.999
	NaviCell	57.91	28.48	6.96			NaviCell	56.52	23.91	19.56	
3	DxH 800	56.00	30.60	7.30	0.985	13	DxH 800	58.20	29.30	8.70	0.994
	NaviCell	49.58	35.29	10.92			NaviCell	64.28	25.71	7.87	
4	DxH 800	44.60	35.30	11.40	0.911	14	DxH 800	41.20	46.20	9.90	0.962
	NaviCell	44.29	30.71	23.57			NaviCell	47.62	41.67	10.71	
5	DxH 800	65.40	24.10	7.00	1.000	15	DxH 800	67.30	17.70	12.40	0.991
	NaviCell	65.33	23.71	7.55			NaviCell	69.90	21.36	6.80	
6	DxH 800	58.20	25.90	7.80	0.999	16	DxH 800	79.20	11.00	9.00	1.000
	NaviCell	60.00	25.45	9.09			NaviCell	76.92	13.67	9.40	
7	DxH 800	48.80	39.20	6.00	0.940	17	DxH 800	62.80	27.70	8.60	0.976
	NaviCell	54.8	30.46	9.30			NaviCell	58.46	33.84	4.61	
8	DxH 800	47.00	41.90	7.40	0.981	18	DxH 800	87.20	5.50	6.60	1.000
	NaviCell	45.60	34.95	11.65			NaviCell	89.69	4.12	6.19	
9	DxH 800	42.60	43.80	7.20	0.998	19	DxH 800	45.30	41.30	7.60	0.978
	NaviCell	42.00	41.18	15.29			NaviCell	48.65	36.49	9.46	
10	DxH 800	87.10	6.50	6.10	1.000	20	DxH 800	57.30	29.50	10.50	0.979
	NaviCell	89.25	5.38	3.22			NaviCell	63.04	23.91	13.04	

## Data Availability

Data underlying the results presented in this paper are not publicly available at this time but may be obtained from the authors upon reasonable request.

## Supplementary Materials

The following supporting information can be downloaded at: https://www.mdpi.com/article/10.3390/bios12020047/s1, Figure S1: Schematic of the developed protocol that combines magnetically activated cell sorting (MACS) and the differential density method to selectively isolate three types of leukocytes in blood and obtain samples with high purity and concentration; Figure S2: Shadow images of neutrophils separated using magnetic beads, helper T cells, cytotoxic T cells, and monocytes separated using Ficoll solution and magnetic beads.
