# Supplementary material for: Field-Portable Leukocyte Classification Device Based on Lens-Free Shadow Imaging Technique
Source: Biosensors (Basel). 2022 Jan 18;12(2):47. doi: 10.3390/bios12020047 (PMC8869521; doi:10.3390/bios12020047)
Supplement: Supplementary file 1 [file biosensors-12-00047-s001.zip › biosensors-1554297-supplementary.pdf]

biosensors

MDPI

Article

Field-Portable Leukocyte Classification Device Based on Lens-Free Shadow Imaging Technique

Dongmin Seo [1,2,†], Euijin Han [1,†], Samir Kumar [1], Eekhyoung Jeon [3], Myung-Hyun Nam [4], Hyun Sik Jun [3,*] and Sungkyu Seo [1,*]

[1] Department of Electronics and Information Engineering, Korea University, Sejong 30019, Korea; dseo@kriso.re.kr (D.S.); haan919@korea.ac.kr (E.H.); skumar@korea.ac.kr (S.K.)
[2] Maritime Safety Research Division, Korea Research Institute of Ships & Ocean Engineering, Daejeon 34103, Korea
[3] Department of Biotechnology and Bioinformatics, Korea University, Sejong 30019, Korea; uuu8345@korea.ac.kr
[4] Department of Laboratory Medicine, Anam Hospital, Korea University College of Medicine, Seoul 02841, Korea; yuret@korea.ac.kr
* Correspondence: toddjun@korea.ac.kr (H.S.J.); sseo@korea.ac.kr (S.S.); Tel.: +82-44-860-1411 (H.S.J.);
+82-44-860-1427 (S.S.); Fax: +82-44-860-1598 (H.S.J.); +82-44-860-1585 (S.S.)
† These authors contributed equally to this work.

biosensors

MDPI

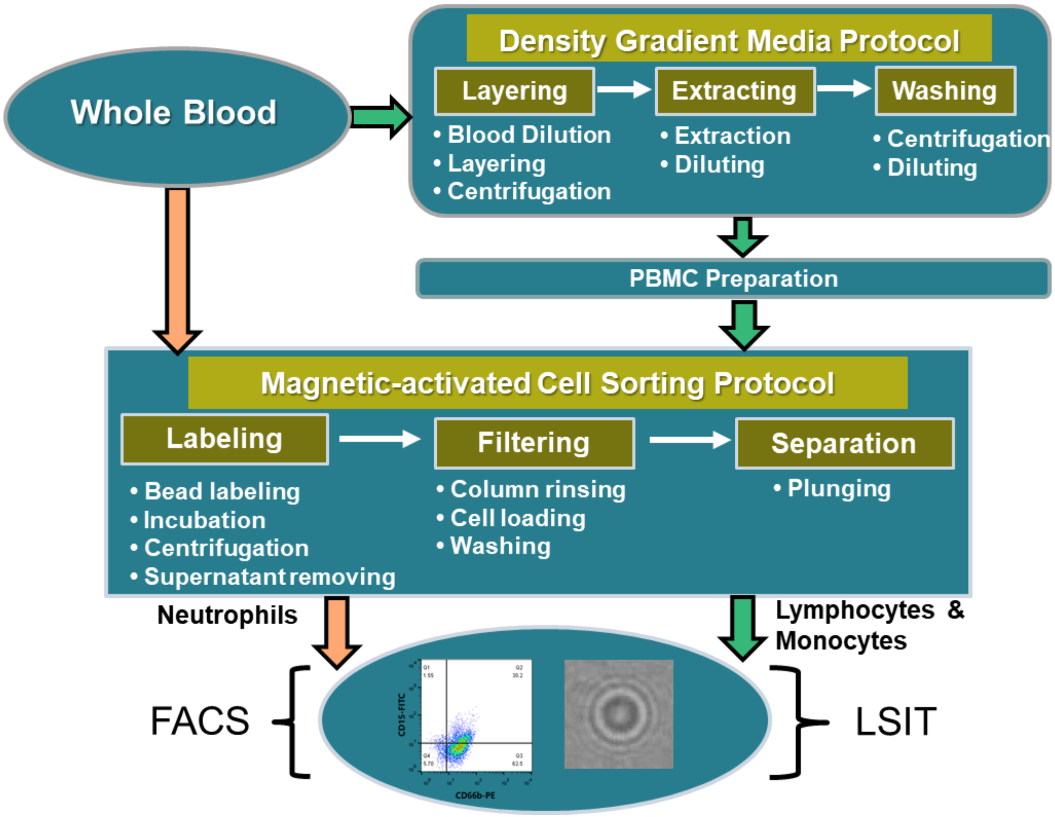

Figure S1. Schematic of the developed protocol that combines magnetically activated cell sorting (MACS) and the differential density method to selectively isolate three types of leukocytes in blood and obtain samples with high purity and concentration.

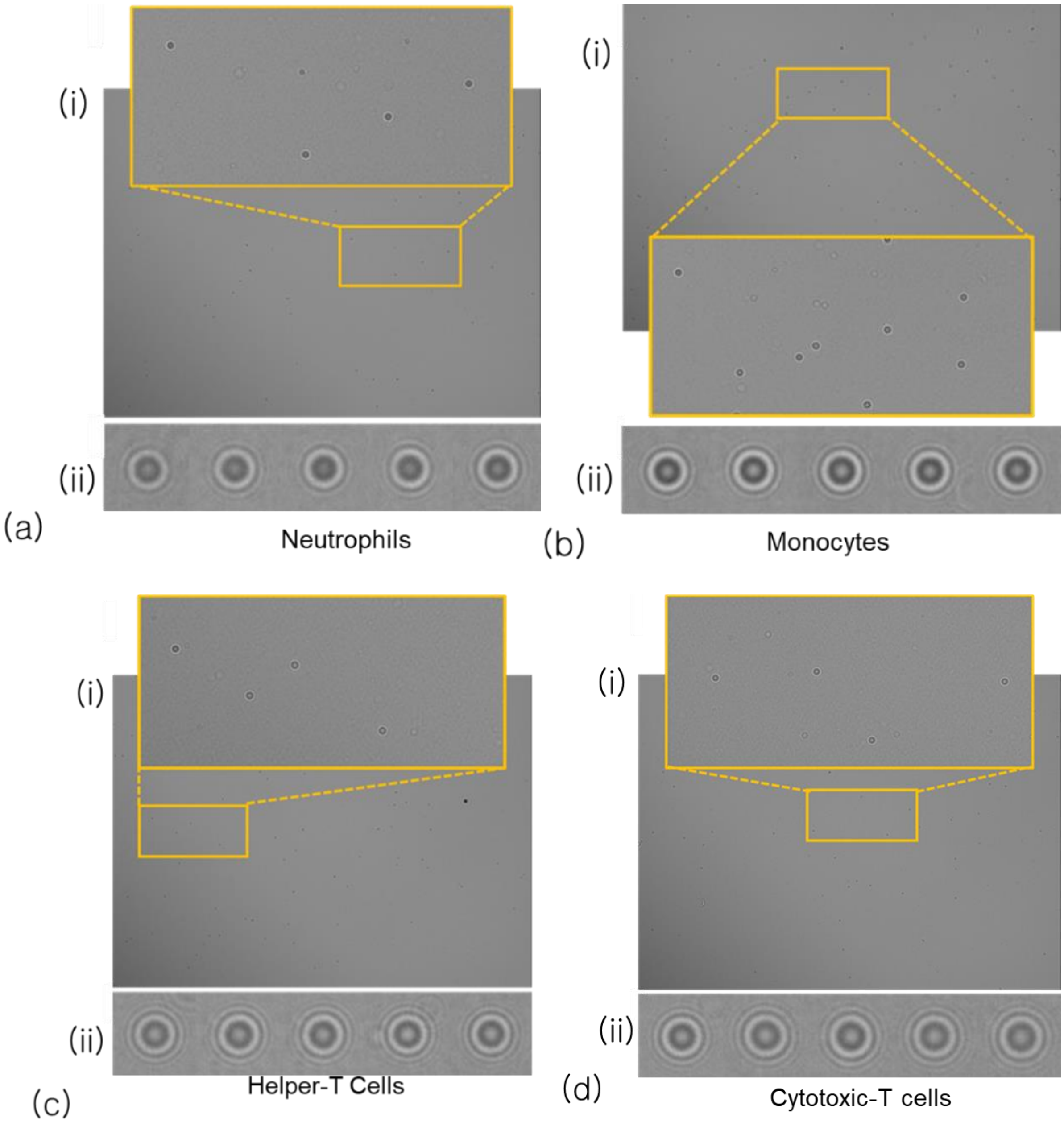

Figure S2. Shadow images of (a) neutrophils separated using magnetic beads, (b) helper T cells, (c) cytotoxic T cells, and (d) monocytes separated using Ficoll solution and magnetic beads. (i) and (ii) are the actual and magnified (200%) shadow images, respectively.
